# Two-Dimensional Nanomaterials beyond Graphene for Biomedical Applications

**DOI:** 10.3390/jfb13010027

**Published:** 2022-03-09

**Authors:** Maryam Derakhshi, Sahar Daemi, Pegah Shahini, Afagh Habibzadeh, Ebrahim Mostafavi, Ali Akbar Ashkarran

**Affiliations:** 1 Precision Health Program and Department of Radiology, Michigan State University, East Lansing, MI 48824, USA; derakhshi_maryam@yahoo.com (M.D.); p_shahini@yahoo.com (P.S.); 2 Department of Chemistry, University of California Davis, One Shields Avenue, Davis, CA 95616, USA; sdaemi@ucdavis.edu; 3 Department of Chemistry, University of British Columbia, Vancouver, BC V6T 1Z1, Canada; afagh@chem.ubc.ca; 4 Stanford Cardiovascular Institute, Stanford, CA 94305, USA; ebimsv@stanford.edu; 5 Department of Medicine, Stanford University School of Medicine, Stanford, CA 94305, USA

**Keywords:** two-dimensional nanomaterials, bioelectronics, imaging, drug delivery, tissue engineering, regenerative medicine

## Abstract

Two-dimensional (2D) nanomaterials (e.g., graphene) have shown to have a high potential in future biomedical applications due to their unique physicochemical properties such as unusual electrical conductivity, high biocompatibility, large surface area, and extraordinary thermal and mechanical properties. Although the potential of graphene as the most common 2D nanomaterials in biomedical applications has been extensively investigated, the practical use of other nanoengineered 2D materials beyond graphene such as transition metal dichalcogenides (TMDs), topological insulators (TIs), phosphorene, antimonene, bismuthene, metal–organic frameworks (MOFs) and MXenes for biomedical applications have not been appreciated so far. This review highlights not only the unique opportunities of 2D nanomaterials beyond graphene in various biomedical research areas such as bioelectronics, imaging, drug delivery, tissue engineering, and regenerative medicine but also addresses the risk factors and challenges ahead from the medical perspective and clinical translation of nanoengineered 2D materials. In conclusion, the perspectives and future roadmap of nanoengineered 2D materials beyond graphene are outlined for biomedical applications.

## 1. Introduction

Two-dimensional nanomaterials (2DNMs) received remarkable attention within the scientific communities as a class of new nanomaterials in recent years. 2DNMs, which range from nanometer to micrometer scales, have one or a few atomic thicknesses and are one of the most promising materials for biomedical applications owing to their special structure and unique properties [1,2]. A critical milestone in the expansion of 2DNMs was the discovery of graphene [3]. Although graphene and graphene-based materials are not the main focus of this review, it is noteworthy that the huge amount of research in the field of graphene paved the way for innovation of other functional 2DNMs (e.g., MXenes). Graphene is the name for a single-layer sheet of carbon atoms that forms a hexagonal honeycomb lattice through sp^2^ bonds, which was first discovered by Andre Geim and Konstantin Novoselov in 2004 using mechanical exfoliation of graphite [4]. Graphene along with its derivatives (graphene oxide (GO) and reduced GO (rGO)) can easily be functionalized by other nanoparticles or functional oxygenated groups due to their special physicochemical properties and as a result have drawn extensive attention related to biomedical applications [5,6].

The significant progress of graphene-based materials in the past decades led to the emergence of a new class of functional 2DNMs such as transition metal dichalcogenides (TMDs), topological insulators (TIs), phosphorene, transition metal oxides (TMOs), black phosphorus (BP), layered double hydroxides (LDHs), hexagonal boron nitride (h-BN), antimonene (AM), bismuthene, metal–organic frameworks (MOFs) and nitrides and carbonitrides (MXenes) [7,8,9,10]. 2DNMs are broadly used in bioelectronics, imaging, drug delivery, tissue engineering and regenerative medicine due to their exotic physicochemical properties, biocompatibility, biodegradability, surface functionality, and plasticity compared to their bulk form [2,11,12,13]. 

**Figure 1:**
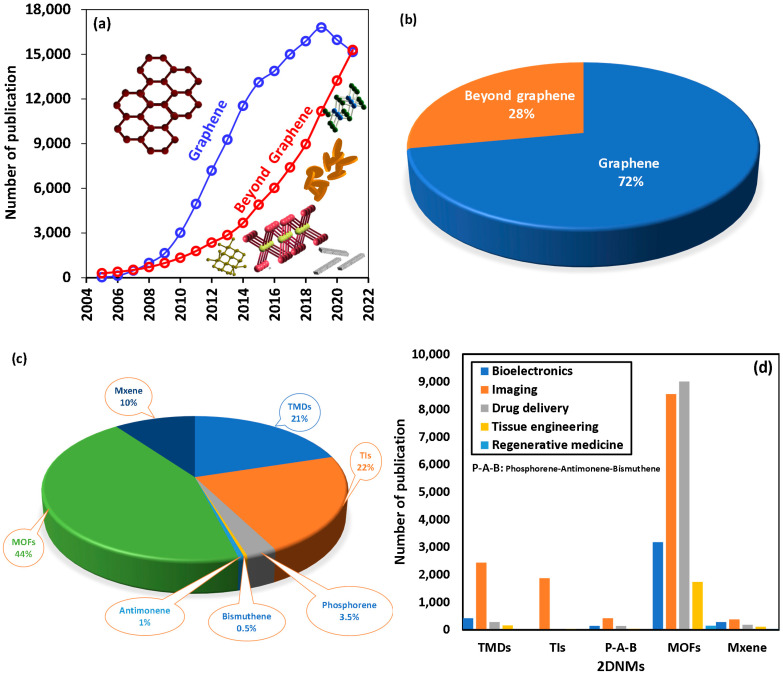
Trend of publications in 2DNMS: (**a**) number of publications of 2DNMS; (**b**) pie chart showing the contribution of the graphene and beyond graphene 2DNMs in publications; (**c**) pie chart illustrating the different contribution of 2DNMs beyond graphene in publications; and (**d**) contribution of each specific 2DNMS beyond graphene in various biomedical applications.

An extended search using the Scopus database (date of search: 20 December 2021) using keywords including transition metal dichalcogenides (TMDs), topological insulators (TIs), phosphorene, antimonene, and bismuthine (P-A-B), metal–organic frameworks (MOFs) and MXenes reveal that 2DNMS beyond graphene have attracted much attention withing the scientific communities (Figure 1). Although a major portion of the publications on 2D materials is related to graphene and graphene-based materials [14,15,16], publications on 2DNMs beyond graphene have grown significantly following the same exponential behavior of graphene, as the first introduced 2DNMs (Figure 1a,b). More specifically MOFs, TMDs, Tis, and very recently Mxenes have shown to have great potential for future biomedical applications (Figure 1c). Statistical analysis of the published papers demonstrated that among various biomedical applications of 2DNMs beyond graphene, imaging, drug delivery, and regenerative medicine have attracted the most attention (Figure 1d). 

Such 2DNMs have two outstanding features compared to other nanostructures: (i) the optical properties of 2DNMs can be easily controlled by changing the number of layers or combining them with other plasmonic nanoparticles; (ii) by modifying surface chemistry of 2DNMs through functionalization, they can be used to deliver specific therapeutic drugs [17]. Furthermore, superior performances (e.g., inherent quantum effects and larger surface area) of 2DNMs compared to other conventional nanostructures made them an appropriate alternative in nanomedicine and various biomedical applications [12]. Moreover, 2DNMs are known as the thinnest materials, which means they have the highest specific surface area among all available materials. On the other hand, the high specific surface area of 2DNMs makes them have large reservoirs and anchoring sites for effective loading and delivery of therapeutic agents [18]. Therefore, 2DNMs can be used in drug delivery systems due to the adsorption of large numbers of drug molecules and excellent control over release kinetics [10]. 

The thinness of the 2DNMs also allows them to be useful for a variety of optical applications (e.g., imaging), since the thinness allows them to respond quickly to external signals [19,20]. In addition, it is reported that 2DNMs have plasmonic properties in the near-infrared region which make them an appropriate photothermal agent for photothermal therapies [17]. Furthermore, the extraordinary mechanical strength and low toxicity of 2DNMs result in a high potential for tissue engineering applications. In fact, the combination of such unique optical and mechanical properties increases the biocompatibility of scaffolds and osteogenic differentiation, which are two brilliant features for future tissue engineering applications [21].

In this review, the unique properties and the potentials of 2DNMs beyond graphene for various biomedical applications are highlighted. Furthermore, the opportunities and challenges ahead of various biomedical applications as well as biosafety and toxicity evaluations of 2DNMs beyond graphene are discussed. 

## 2. Structures of 2DNMs beyond Graphene

**Figure 2:**
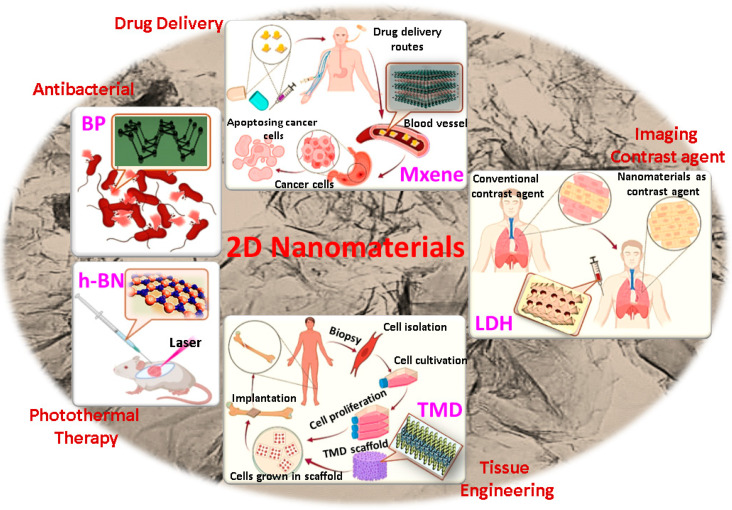
2DNMs beyond graphene. Schematic of 2D layered nanomaterials beyond graphene and their potential biomedical applications.

The physicochemical properties of nanomaterials strongly depend on their atomic structure and arrangement. Although the crystal structure and atomic composition of 2DNMs differ from each other, they can be classified into two categories: layered and nonlayered 2DNMs [22]. In layered nanostructures, the atoms present in a layer are bonded together by strong chemical bonding, while the weak van der Waals interaction between the layers causes the layers to stick to one another. One of the well-known representatives of 2D layered nanomaterials is graphite, in which graphene layers are stacked together [23]. Other graphene-analogous 2DNMs include TMDs, BP, LDHs, h-BN and MXenes (Figure 2). As shown in Figure 2, these nanostructures are honeycomb-shaped, but the location of adjacent atoms in the top and bottom layers of 2DNMs is different. In contrast to layered nanomaterials, nonlayered nanomaterials crystallize in three dimensions through atomic or chemical bonds to form polymers and metal chalcogenides, which are bulky crystals [10]. 

**Figure 3:**
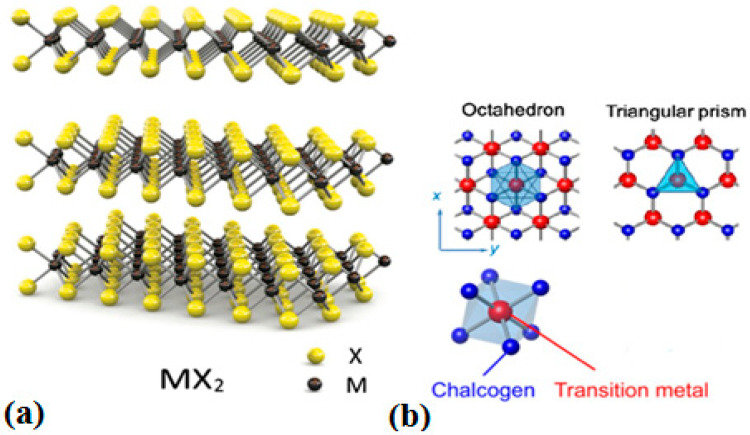
Overall structure of TMDs: (**a**) Three-dimensional representation of TMDCs structure; and (**b**) Top view of monolayers constructed from octahedral and triangular prismatic coordination (Reprinted with permission from ref. [22]. 2019, Elsevier).

TMDs are an important class of 2DNMs with the chemical formula of MX_2_. In an X–M–X layered structure of TMDs, M is a transition metal element of groups IV-VII (e.g., Ti, Zr, Hf, V, Nb, Ta, Mo, W, Tc and Re) and X is the group 16 elements of the periodic table (e.g., S, Se and Te) [24]. Each TMDs layer has a structure of three atomic layers, in which one transition metal layer is sandwiched between two chalcogen layers, as shown in Figure 3. The M-X atoms are strongly attached via covalent bonding, and the layers are stacked by weak van der Waals bonds that closely resemble graphite [25]. TMDs have a triangular and octagonal structure in which the thickness of each monolayer is approximately 0.7 nm [22]. Every transition metal atom is bounded by six chalcogen atoms that form triangular and octagonal structures (Figure 3). The large band gap (i.e., >1 eV) of TMDCs make them an ideal material for modern bioelectronics [26]. 

**Figure 4:**
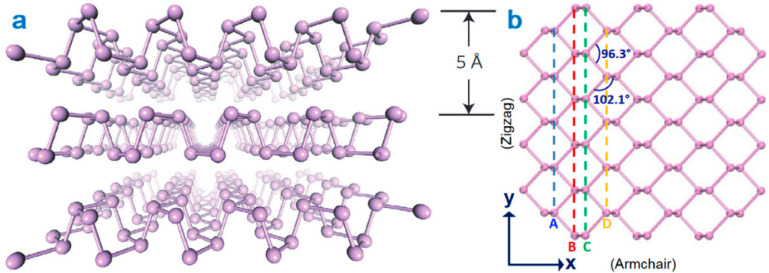
Crystalline structure of BP: (**a**) atomic structure of BP; (**b**) top view of the lattice of single-layer BP. (Reprinted with permission from ref. [29]. 2019, Elsevier).

BP, the most stable allotrope of phosphorus, is another class of 2D material that is expected to be promising candidate for a wide range of biomedical applications. BP was first synthesized by Bridgman in 1914, and after a century, once again attracted the attention of researchers as a promising member of the 2DNMs family [27]. In contrast to TMDs with indirect bandgaps, BP has a direct bandgap of about 0.3 eV (bulk structure) to 2.0 eV (monolayered structure). Therefore, due to broadband absorption from the visible to mid-infrared region of electromagnetic waves, BP is suitable for many optoelectronic applications [28]. Each layer of the phosphorus atoms in BP is connected to three adjacent atoms, forming a stable loop structure with an interlayer distance of ~5 Å (Figure 4). There are two types of P-P bonds inside the crystal lattice: (i) the P–P bond in the same plane with bond length 2.224 Å and bond angle with 96.3°; (ii) P-P bond in a different plane with bond length 0.2244 nm and bond angle with 102.10° [29]. The single layers of BP show two kinds of P–P bonds with different bond lengths, thereby BP is divided into armchair and zigzag type according to the shape. This causes the anisotropy of the crystal structure of BP (Figure 3b) [30]. 

Anisotropy of BP crystal structure is associated with the anisotropy of electrical and optical properties along three crystal axes which can be used for various near and mid-infrared optoelectronic applications, such as photodetectors and modulators [29]. 

**Figure 5:**
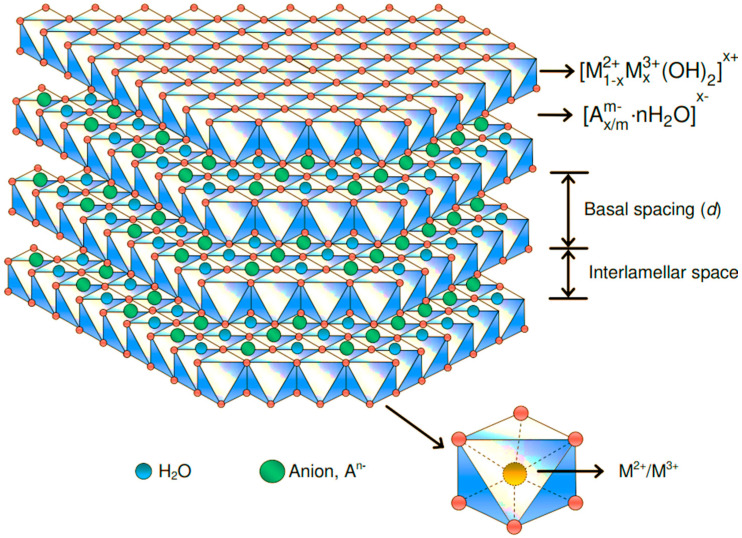
The overall structure of LDH: Schematic representation of layered double hydroxides (LDH) structures (Reprinted with permission from ref. [32]).

LDHs with a two-dimensional layered structure and previously known as anionic clays, can be represented by the general chemical formula [M(1−X)2+MX3+(OH)2]X+(An−)xn·mH2O, where M^2+^ and M^3+^ represent the divalent and trivalent metal cations, respectively; A^n−^ is an interlayer n-valent anion (e.g., Cl^−^, OH^−^, NO^3−^, SO_4_^2−^ and organic anions); the value of x is equal to the molar ratio of M^2+^/(M^2+^ + M^3+^) and m is the number of crystal water molecules [31]. LDHs are structurally similar to brucite in which a layer of cationic metal atoms is sandwiched between hydroxide layers (Figure 5). 

The presence of positively charged sites on the surfaces of layers in LDHs leads to noncovalently bound anionic drug molecules and genetic material, making LDHs ideal materials for drug delivery applications. Another advantage of LDH nanosheets for drug delivery is their ability to control the release of pharmaceutically active compounds due to their biocompatibility and alkaline character [33].

**Figure 6:**
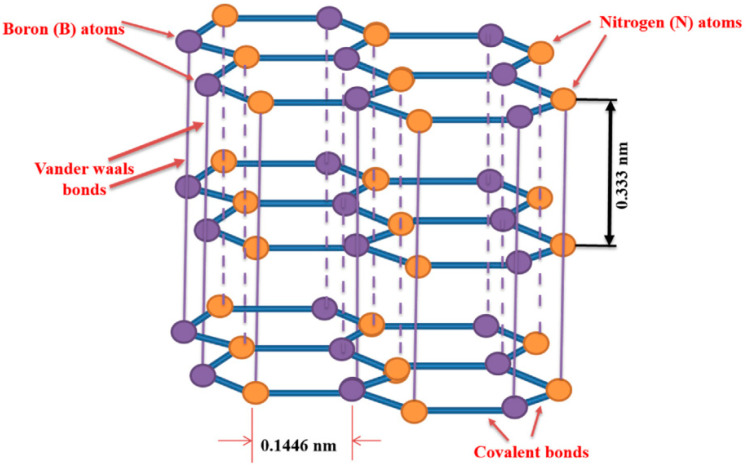
Atomic structure of h-BN 2D materials; Schematic diagram of the h-BN structure (Reprinted with permission from ref. [35]. 2020, Elsevier).

Hexagonal boron nitrides (h-BN) which are another class of 2DNMs which are structurally more similar to traditional graphene and commonly known as the ‘‘white graphene”. The building block of monolayer h-BN exhibits a hexagonal structure which consists of an equal number of boron and nitrogen atoms (Figure 6) [34]. Due to the difference in electronegativity, the electrons in the B-N bonds of h-BN move toward N atoms, so these bonds have ionic properties compared to covalent C-C bonds in graphene [35]. 

It is reported that polarization of the BN bond significantly affects physicochemical properties of h-BN, including chemical conjugation (any nucleophilic group and electrophilic group can target the B and N atom, respectively) and band gap energy (band gap~ 5–6 eV) [36]. Therefore, due to these properties, h-BN has demonstrated potential in the biomedical applications such as wound healing and bone tissue regeneration [37]. 

**Figure 7:**
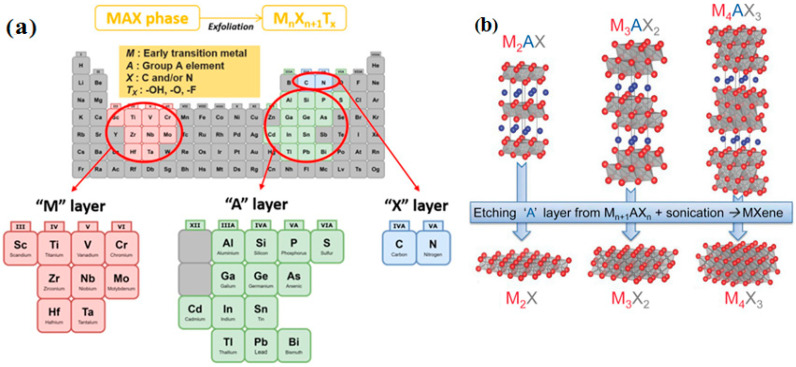
Crystalline structure of MXene: (**a**) General element composition of MAX phase and MXene, (Reprinted with permission from ref. [39]. 2021, BMC); (**b**) MXene chemistry (Reprinted with permission from ref. [40]. 2020, Elsevier).

Recently, MXenes as a novel group of interesting 2DNMs were discovered by Naguib et al. who exfoliated 3D titanium aluminium carbide using hydrofuoric acid and produced 2D titanium-carbide layers. MXenes are a family of transition metal carbides and nitrides that are produced by exfoliating their 3D precursors. The 3D precursors for MXenes are called MAX phases, which have a general formula in the form of M_n+1_AX_n_, where M is a transition metal, A is an A-group element (mostly main group IIIA or IVA), X is carbon or nitrogen and n = 1, 2 or 3 (Figure 7) [38]. M–X bonds are stronger than M–A bonds, and A layers are more chemically active than M–X layers. Therefore, M_n+1_X_n_ layers are created by the elimination of A layers through selective strong acid etching. Finally, the final chemical formula of MXenes is M_n+1_X_n_ T_x_, where T_x_ is the surface functional groups (e.g., fluorine (–F), hydroxide (–OH) and oxygen (–O) groups) and x is the number of surface functionalities [39]. In recent years MXenes have been used as novel photothermal therapy agents to treat mouse breast cancer cells due to their unique properties [40]. 

## 3. Design and Synthesis of 2D Nanomaterial

The synthesis/fabrication of 2D materials can be mainly categorized as two top-down and bottom-up methods. The top-down method relies on mechanical forces to destroy the weak van der Waals interaction between layers to obtain a single or multilayer nanometer sheets, such as mechanical cleavage and liquid exfoliation [1,10,41]. 

In the other hand, the bottom-up methods are usually based on chemical conversion under certain conditions to form ultrathin 2DNMs, including chemical vapor deposition (CVD) and wet-chemical synthesis (e.g., hydro-/solvo-thermal, self-assembly of crystals and soft colloidal synthesis) [42,43]. However, the CVD method requires high vacuum, high temperature and special substrates. Wet-chemical synthesis is another typical bottom-up method that is emerging as a very promising alternative to produce ultrathin 2DNMs. Wet-chemical synthesis leads to the high-yield, low-cost and massive production of ultrathin 2D nanosheets in the solution phase, which is suitable for industrial production [44]. The wet-chemical method is under the influence of reaction parameters such as reaction temperature, reaction time, concentration of precursors and solvents. These parameters are effective in controlling the size and shape of 2DNMs. Several main wet-chemical methods used for the synthesis of 2DNMs include hydro/solvothermal synthesis, self-assembly of nanocrystals, and soft colloidal synthesis. The process involving water or organic solvent is called a hydrothermal or a solvothermal process, respectively. In the hydro/solvent method, researchers use water or organic solvent as the reaction medium in a sealed vessel, in which the reaction temperature is higher than the boiling point of the solvent [45]. In the first step, the precursors are mixed in a solvent and then sealed in an autoclave. In the next step, the autoclave as a closed system is heated to a temperature above the boiling point of the solvent. The autoclave enhances the temperature and pressure of the solution and leads to crystallization of the soluble material at high pressure. This method has been widely used to achieve high purity and homogeneously ultrathin 2DNMs [45]. Self-assembly of nanocrystals should be considered as another efficient way to produce 2DNMs due to the development of synthesis technologies and surface modification of nanocrystals. Generally, the fusion of low-dimensional nanocrystals such as nanoparticles and nanowires during the assembly process leads to the formation of 2DNMs, in which presynthesized nanocrystals as basic building blocks automatically organize with each other by noncovalent interactions such as van der Waals interactions and electrostatic interactions [45]. The result of this method is 2D nanosheets that have been used in biomedical applications. 

**Figure 8:**
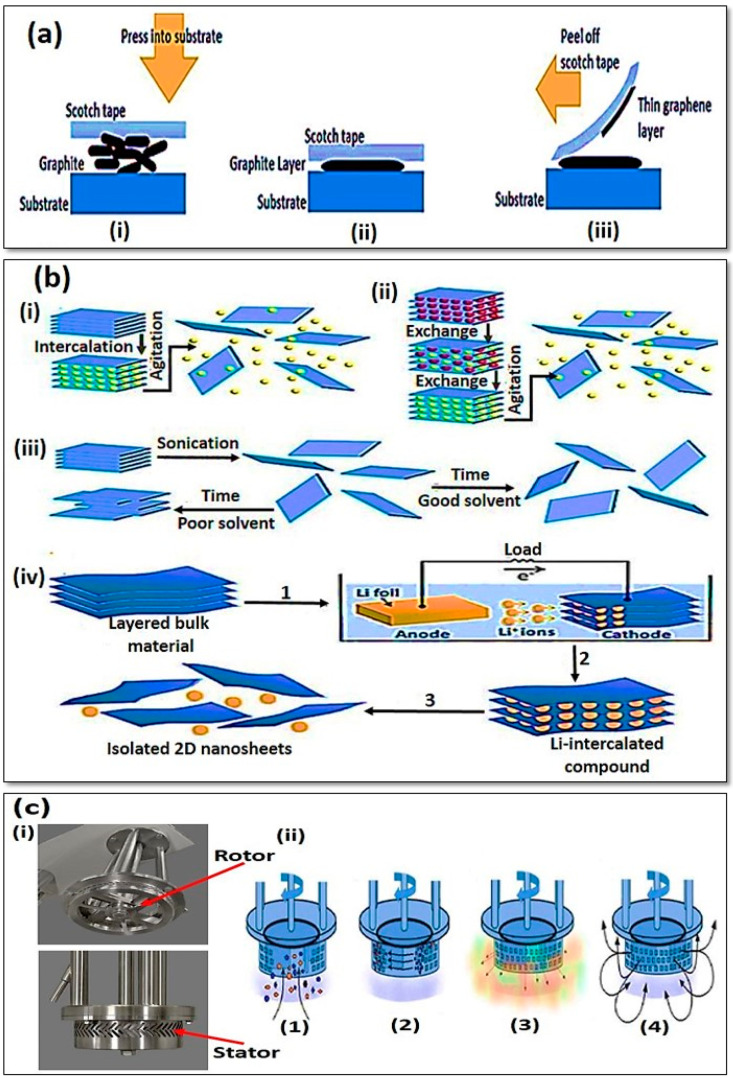
Exfoliation and mechanical approaches for 2DNMs preparation: (**a**) Schematic representation of the scotch tape exfoliation method to produce graphene. Steps (i) to (iii) are repeated until the graphite is reduced to a thin layer of graphene on the substrate (Reprinted with permission from ref. [51]. 2020, MYU K.K.); (**b**) Schematic representation of the main liquid exfoliation mechanisms: (i) ion intercalation; (ii) ion exchange; (iii) sonication-assisted exfoliation; (iv) electrochemical exfoliation (Reprinted with permission from ref. [25]. 2017, Elsevier); (**c**) (i) Images of the shear mixer; (ii) graphic representation of shearing steps (Reprinted with permission from ref. [52]. 2018, American Chemical Society).

Mechanical exfoliation, or the so-called “scotch-tape method”, is a conventional process to peel off bulk layered material into single or several layers of nanometer sheets by using the adhesive force of scotch tape [22,46]. In this type of exfoliation method, thin 2DNMs are obtained by weakening the van der Waals interaction between the layers without breaking the in-plane covalent bonds of each layer [47]. In 2004 Novoselov and co-workers obtained the first single layer graphene by a mechanical cleavage method from small crystals of highly oriented pyrolytic graphite (Figure 8a) [47]. Since then, this method has been widely used for exfoliation of various kinds of 2DNMs such as h-BN and TMDs [22]. Mechanically exfoliated 2DNMs have high crystal quality (few defects) and clean surfaces because no chemical impurities are introduced in this procedure. However, low production yield and uncontrollable thickness are disadvantages of this method, and the method is not suitable for scale up applications [48]. Liquid-phase exfoliation (by mechanical force such as sonication, chemical or electrochemical exfoliation and shear exfoliation) is another top-down strategy to obtain 2DNMs using external forces in liquid media, which basically involves two processes (intercalation and sonication) (Figure 8b) [22,49]. Direct liquid exfoliation, also called sonication-assisted exfoliation in organic solvents, ionic liquids and solvents or aqueous surfactant solutions, can be used to extract individual layers. In direct liquid exfoliation, the bulk crystal is firstly dispersed in a specific medium such as dimethylformamide (DMF) or N-methyl-pyrrolidone (NMP). Under sonication, the ultrasonic wave can generate cavitation bubbles and high-speed microjets through hydroxyl radical or pyrolysis reaction on the surface of the crystal [1]. Then, tensile forces appear to remove the van der Waals force between the layers. On the other hand, solvent molecules can stabilize exfoliated nanosheets and prevent their aggregation by binding to the surface of nanosheets. Therefore, suitable ultrasonic intensity, ultrasonic time and power, temperature and solvent type are critical parameters affecting the production of single-layer or few-layer 2DNMs [50]. Although liquid exfoliation reduces some disadvantages of mechanical exfoliation, in most cases excess chemicals from the solvents remain or defects are created by ultrasound, which affect the final quality and physicochemical properties of 2DNMs [9]. 

The thinning process of bulk crystals can also be achieved with the help of a chemical or an electrochemical exfoliation method. The chemical exfoliation process involves the use of ultrasound to insert intercalators between the layers of bulk crystals (Figure 8b). Intercalators include acid/base compounds, oxidizing agents, functional molecules and inorganic salts, of which the most common are organometallic compounds such as butyl lithium [53]. In the procedure of electrochemical exfoliation, bulk and intercalant materials are placed at the anode and cathode pole of the battery, respectively. Then, by applying an electrochemical bias, the ions can be intercalated into an interlayer of bulk material, which causes the weakening of the van der Waals force between the layers [54]. In most cases, hydrogen gas is produced due to the interaction of intercalated ions with water, which can help to separate the adjacent layers [47]. Finally, high-yield 2D nanosheets can be obtained after the centrifugation process. Bath or probe ultrasonication produces 2DNMs at the scale of 10 or 100 mg, while more scales are needed to move towards commercialization [27].

Another recently emerging method for producing 2DNMs is the use of shear force-assisted exfoliation, which can be scaled up to the industrial level. The shear force apparatus has a rotating blade constituting a rotor and a stator (Figure 8c). In this apparatus the rotating blade can produce high shear rates in liquid containing layered bulk crystals [43]. The fabrication process of 2DNMs using a shear exfoliation method includes: (i) rotation of the rotor to create a strong pressure gradient that directs the dispersion from the bottom to the center of the container; (ii) the centrifugal force to move the particles to the gap between the rotor and the stator (particles are milled here); (iii) shear exfoliation of particles due to the high-speed rotation and then a push out of the small holes in the stator; and (iv) draw the materials into the container to maintain the mixing cycle. Based on a high-shear rotor-stator mixer, Woomer et al. used shear-assisted exfoliation to exfoliate BP bulk crystals into large-scale of few-layer nanosheets [55]. They have shown that the band gap of BP increased from 0.33 ± 0.02 eV in bulk to 1.88 ± 0.24 eV in bilayers, a range that is larger than any other 2D material.

In recent years, chemical vapor deposition (CVD) has emerged as the most promising bottom-up approach to prepare 2DNMs. In this technique, the vapor-phase active precursors can react or decompose on the desired substrate at high temperature and low pressures to form large-scale ultrathin 2DNMs [56]. It is worth pointing out that precursors, substrates, catalysts, temperature, and atmosphere play a key role in the quality of the final products in this technique. Compared to other methods, CVD produces 2DNMs with the highest level of control, high purity and crystal quality and the least defects [56]. For instance, Lee et al. successfully prepared single-layer MoS_2_ films onto amorphous SiO_2_ substrates by the CVD process with MoO_3_ and S powders as the reactants at 650 °C. Their findings reveal that the on-off current ratio exceeds 10^7^, and the mobility is up to 1.2 cm^2^/V·s, which is comparable to an exfoliated MoS_2_ monolayer fabricated without high k-dielectrics [57].

## 4. General Properties of 2DNMs

### 4.1. Physical Properties

The reduced dimensionality of 2DNMs results in unique characteristics that make them different from their bulk counterparts, yet similar to each other. Since the surface of 2DNMs plays a crucial role in their properties, manipulating the surface enables fine tuning of different properties in different fields of research and interest such as lubricants, thermoelectronics, catalysis, energy storage application, FET devices and specifically biomedical applications [58]. In fact, the exclusive structure of 2DNMs can give rise to unique characteristics such as high surface area, high modulus and strength which play a key role in biomedical applications.

2DNMs stemming from restriction of bulk components to grow in a certain direction result in their interesting physicochemical properties. While preserving their large longitudinal dimension, 2DNMs can still maintain their atomic thickness which leads to ultra-high specific surface area and high ratio of exposed atom on a surface. Such a high surface area per volume of 2DNMs makes them different from conventional nanomaterials with potential biomedical applications (e.g., drug delivery and nucleic acid carriers) [59,60]. The ultra-thinness of 2DNMs combined with their intrinsic strong in-plane covalent bonds also plays an important role in the flexibility of these materials. Within a layer of 2DNMs, atoms are tightly bound to each other by covalent or ionic bonds which results in their high strength [61].

The flexibility and strength can be tuned by altering the in-plane bonding as well as the thickness of these materials, enabling each of them to be used in a particular application such as differentiation of stem cells, tissue engineering, monitoring intracranial brain injury and controlled drug delivery [62,63,64]. For instance, in a study clay-based 2DNMs were added to Poly(lactic-co-glycolide) (PLG) to result in enhanced fracture strength, toughness and elongation from 7% to 210%. Tuning the physical properties such as thickness and strain also plays an important role in controlling the other properties of 2DNMs such as their optical and electronic characteristics which enable them to be used in a variety of therapeutics.

### 4.2. Electronic Properties

It is well documented that 2DNMs have strong in-plane covalent bonds, however, their interlayer interactions are low or zero (i.e., single-layer nanosheets). Various classes of 2D materials show different electronic properties ranging from semimetals (i.e., graphene, which makes them an appropriate alternative for tissue engineering and biosensing applications such as prosthetic skins) to insulators (h-BN and clay-based nanomaterials) and a variety of semiconductors (i.e., TMDs, TMOs and xenes) [65]. Tuning the conductivity of 2DNMs by controlling their structure results in a variety of biomedical applications such as nanopore sequencing and FET-based biosensing, sequence-specific transistors, biosensing, drug delivery, PDT and bioimaging [19]. Moreover, due to different extents of defects, doping or synthetic methods could also be applied to change the electronic properties. Some of the most important aspects of 2DNMs such as energy level, scattering and excitation depend on external influences such as doping and thickness of the layer. Defects in general would cause lower conductivity since the free movement of electrons in a pristine plane of 2D materials is halted by adatoms. This change in electronics can be utilized in detecting signal molecules such as reducing agents or oxides. Under external stimulation (i.e., light excitation) electron movements in conducting and valence bands will change which can lead to a reaction of the active electrons with the ambient environment and impose oxidative stress. This feature of 2DNMs can be exhibited in their use in reactive oxygen species (ROS) generation and catalysis which can further be applied in photodynamic therapy (PDT) and biocatalysis [10]. Combining different properties of 2DNMs enables more efficient results than tuning an individual property. Coupling optical and mechanical properties of 2DNMs with electronics leads to their versatile use in optoelectronic and piezoelectricity such as photodiodes, light emitting diodes, phototransistors, etc. [66].

### 4.3. Optical Properties

The optical properties of 2DNMs strongly depend on their absorption or emission of light which is in close relation to their electronic band structure. Spin-orbital interactions play an important role in modifying the optical properties of 2DNMs by changing the bandgap responsible for light absorption. Reduced dimensionality in some classes of 2DNMs shifts their indirect bandgap to a large direct bandgap (e.g., TMDs) which results in a significant change in optical properties. In fact, large bandgap results in both light absorbing and light emitting properties and small bandgaps can only possess light-absorbing properties and are incapable of emitting light [67]. The ability to interact with light in a wide range of NIR to ultraviolet renders 2DNMs suitable for a plethora of applications such as bioimaging and biosensing. 

Semiconducting 2D (e.g., TMOs) nanomaterials can be used as photosensitizers due to their large optical absorption in UV regions to generate singlet oxygen that makes them ideal as PDT and photoacoustic imaging (PAI) agents for selective killing of both bacteria and cancer cells [68]. Tuning the structure such as building nanocomposites based on 2NMs expands their light response to a visible region by inhibiting their electron-hole pair recombination. Due to their high photothermal effect of semiconducting nanosheets under NIR irradiation, they are promising tools in pH/photosensitive drug delivery systems, PTT, PDT and chemotherapy [69].

Excitation at particular wavelengths leads to emissions in some classes of 2DNMs which can be utilized for PLTI that benefits bioimaging and optical-based biosensing. It is reported that emission of 2DNMS can be tuned by trapping defects which makes them favorable toward optoelectronic applications. Some of the 2DNMs also exhibit localized surface plasmon resonance which leads to a useful substrate for high-resolution bioimaging and scattering-based biosensing. 

### 4.4. Chemical Properties

Ultrahigh surface area to volume of 2DNMs compared with other nanostructures provides extensive surface interactions between 2DNMs and therapeutic, diagnostic and theranostic molecules and therefore, unique opportunities for biomedical applications and nanomedicine. It is reported that surface modifications (e.g., with poly(ethyleneglycol) (PEG)), protection (e.g., with poly(vinylpyrrolidone) (PVP)), encapsulation (e.g., with poly(lactic-co-glycolic acid) (PLGA)) and targeting linker functionalization enhances their corresponding biomedical performance such as biocompatibility and target ability [70]. Due to the ultrathin structure, 2DNMs can show rapid response to external stimuli such as pH change which in turn results in controlled release of loaded molecules in desired sites for drug delivery purposes and deep tumor penetration by both extrinsic stimuli (e.g., near-infrared (NIR) light) and intrinsic stimuli (i.e., pH) [59].

Although inherently 2DNMs might not be air or water stable, their physicochemical properties can be easily tuned using surface functionalization [71]. Moreover, versatile tuning of surface chemistry provides a range of selectivity and analysis that determine their use in safe biomedical applications such as attaching to biological markers. The attachment of these markers on the surface of the 2DNMs can change their electronic signals which can be used for ultrasensitive detection of these markers [15,16]. The capability of multifunctionalizations of 2DNMs enables tuning of their properties which results in a versatile biomedical and nanomedicine applications. 

## 5. Biomedical Applications of 2D Nanomaterial

### 5.1. Bioelectronics

Due to their specific geometry and unique physicochemical properties such as high conductivity and flexibility, 2D nanomaterials are appropriate choices for many bioelectronic applications (e.g., wearable sensors). One of the earliest 2DNMs that has been widely investigated in the field of wearable electronics, is graphene nanosheets. In a recent study by Kwon and co-workers, high-aspect ratio functionalized conductive graphene are produced through exfoliation of graphite in ammonium sulfate solution. The obtained ink with defined concentration was used for printing gel-free wireless flexible electrodes for monitoring muscle activities. The all-printed stretchable electrode prepared through so-called “all printed nanomembrane hybrid electronics” technology (p-NHE), with superior compatibility to human skin was used for real-time electromyogram (EMG) recording. The electrode was attached on the three muscles with highest EMG signals: palmaris longus, brachioradialis, and flexor carpi ulnaris, which produced seven signal clusters by sensing motions of fingers. The NHE wearable electrode detects all finger motions for seven different gestures and accuracy as much as 99% through wireless monitoring of EMG signals via Bluetooth, denoting its ability for smart rehabilitation purposes [72].

Graphene can also be attached to biological interfaces with high conductivity (more than 2.6 S·m^−1^) and conformity to make tissue-integrated biointerfaces [67]. In this method, graphene oxide- Polyvinyl alcohol (PVA) hydrogel is first reduced to become conductive reduced graphene oxide (rGO-PVA) before introducing poly (acrylic acid) grafted with N-hydroxysuccinimide (PAA-NHS) ester to make biointerface with wet tissue. The biofunctionality of electrodes in vivo was tested by implanting the electrode in the right atrium and apex of a rat heart, by which stable epicardial ECG with high signal to noise ratio was monitored. It was found that after 14 days the ECG signals became stronger, as a proof of successful biointerface integration [73]. Another noninvasive class of biointerfaces are temporary electronic tattoos for integration to human skin. These cheap accessible biointerfaces can be used to monitor human body status such as recording brain, heart and muscle activities. Kireev et al. developed a facile protocol to obtain graphene electronic tattoos using commercially available CVD-grown graphene without the need for trained labor. They found bilayer graphene electronic tattoos show skin impedance in the range of 8–10 kΩ and sheet resistance as low as 1 kΩ/sq compared to monolayer graphene, suggesting reproducible performance of bilayer graphene with low enough impedance to allow current injection [74].

Although applications of 2DNMs in bioelectronics were first fueled by the emergence of graphene, recently nongraphene 2D materials (e.g., MXenes) have been investigated extensively in biosensors and wearable technologies due to their functionality and physiochemical features that outperform other 2DNMs (i.e., graphene) for biomedical applications. 2D MXenes are conductive thin platforms with high surface area containing chemically reactive sites useful for biofunctionalization. These functional groups make the surface of MXenes hydrophilic compared to other similar 2DNMs, which allows solution preparation of MXenes in aqueous media. Moreover although high conductivity can also be obtained by other materials such as metal NPs and graphene, there are still drawbacks to their application in wearable biocompatible devices [75]. For instance, toxicity of metal NPs is debatable [75], and they normally need a high temperature annealing process which makes them inappropriate for room temperature polymer-based substrates [76]. Graphene, on the other hand, needs to be obtained by graphene oxide reduction [77], which needs high temperature thermal treatment in the presence of hazardous chemicals, ending up in a less hydrophilic layer not good enough for water-based ink solutions [78]. This is why MXenes, with high conductivity and hydrophilicity, are appropriate alternatives for flexible and implantable bioelectronic devices. MXenes have been utilized in facilitating charge transfer between electrode and redox enzymes in the enzymatic biosensor technologies due to their superior electrical conductivity. Therefore, they have been good substitutes for other conducting nanomaterials, thanks to their biocompatibility. 

Black phosphorene (BP) is another class of 2DNMs which can be prepared by exfoliating black phosphorus crystal with high charge carrier mobility, which makes it suitable for electronic devices. However, exfoliated BP has intrinsic instability in ambient conditions, and is prone to degradation and oxidation in air and water environments [79]. One way to passivate BP against corrosion is to combine it with other conductive stable 2D materials such as MXene. In a study performed by Zhu and co-workers [80], titanium carbide MXene (Ti_3_C_2_-MXene) nanohybrid with two-dimensional phosphorene was prepared by electrostatic self-assembly. The nanomaterial then was mounted on laser-induced porous graphene and used as a nonenzymatic electrode for detection of phytoregulator α-naphthalene acetic acid (NAA) residues in agricultural products through a portable wireless electrochemical miniworkstation. The sensor revealed a wide linear range of 0.02–40 μM and a low limit of detection (LOD) of 1.6 nM [80]. Antimonene with similar properties to BP also holds much attention for biosensor application [81]. In a study, antimonene functionalized with supramolecular oligonucleotide and was applied to detect certain DNA sequences and BRCA1 gene mutation caused by breast cancer in real samples [82]. Therefore, this class of 2DNMs with the facile preparation method can potentially be an inexpensive alternative for traditional time-consuming gene assays. 

Nanostructured topological insulators are narrow bandgap 2D materials with high carrier mobility, catalytic activity and delocalized metallic surface states that allow fast interfacial charge dynamic, which leads to highly sensitive electrochemical sensing platforms [83]. Zhao and co-workers synthesized microflakes of Bi_2_Te_3_ with a sensitivity of 4900 μAmM^−1^cm^−2^ and LOD of 10^−8^ molar for electroreduction of hydrogen peroxide, which was greatly enhanced compared to other available metal electrochemical sensors [84].

**Figure 9:**
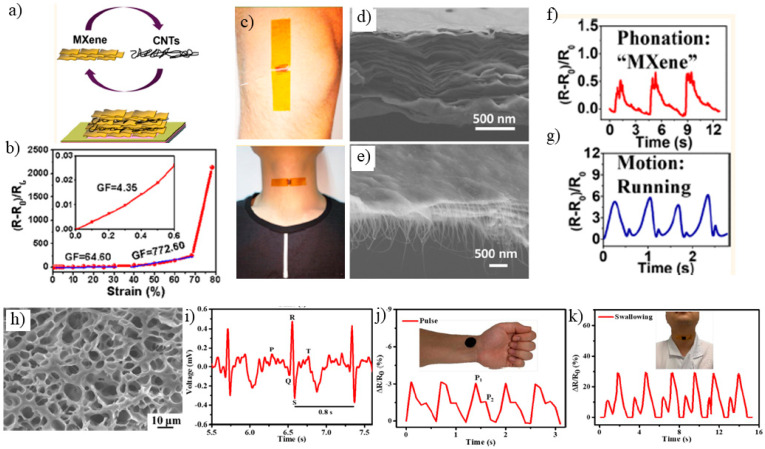
MXene for biosensing applications: (**a**) sandwich-like Ti_3_C_2_T_x_ MXene/CNT layer formation; (**b**) resistance−strain dependence of a Ti_3_C_2_T_x_ MXene/CNT/latex sensor at stretching rate of 5% min^−1^ (inset illustrates the curve considering 0.6% strain); (**c**) digital photograph of a Ti_3_C_2_T_x_ MXene/CNT/latex strain sensor attached to the throat of volunteer; (**d**) Ti_3_C_2_T_x_ MXene flakes; (**e**) cross-sectional SEM image of sensor on throat; (**f**) Ti_3_C_2_T_x_ MXene/CNT layers, (**g**,**h**) responsive curves recorded phonation test of “MXene” word, and detecting human leg movement when running (Reproduced with permission from Ref. [85] Copyright 2018, American Chemical Society); (**h**) SEM images of freeze-dried MXene-PAA-ACC hydrogel; (**i**) typical ECG signals and the recorded relative resistance changes of the strain for (**j**) blood pulse; (**k**) swallowing of a volunteer. (Reproduced with permission from Ref. [86] Copyright 2021, American Chemical Society).

Cai and co-workers prepared sandwich-like Ti_3_C_2_T_x_ MXene/carbon nanotube (CNT) composite using a layer-by-layer air-spray coating of Ti_3_C_2_T_x_ and CNTs. The sensitivity of the device obtained from probing to piezoresistive properties of film through gauge factor relation GF = (R − R_0_)/R_0_ε (ε, R_0_ and R are strain, electrical resistance with no strain, electrical resistance with strain, respectively) revealed a rapid increase at high strain due to disconnection between CNT routes. The sensor was used as a real-time monitoring wearable device to detect physiological movements. It was attached to a human throat while the volunteer was expressing various words with different syllables, and it was able to distinguish between them, indicating the potential application of this sensor for phonation rehabilitation exercises (Figure 9a–g). The sandwiched thin films were able to detect human body deformations with high sensitivity of up to 772.6 in the range of 30% to 130% strain and demonstrated significant stability after more than 5000 cycles [85].

Zhang and co-workers established a new class of wearable strain-sensitive material made of hydrogel polymer-coated MXene (Ti_3_C_2_T_x_) to afford healability and degradability while being an environmentally friendly option for sensing human body motions. The MXene-poly (acrylic acid)-amorphous calcium carbonate (MXene-PAA-ACC) hydrogel was prepared by integrating MXene nanosheets matrix into the blended PAA and ACC network through a facile synthetic method at room temperature. The rapid self-healing property of the MXene-PAA-ACC hydrogel was proven by instant recovery of broken parts, due to the noncovalent interaction between surface termination groups of MXene sheets and the carboxylic groups of PAA and Ca^2+^. The sensitivity of the MXene-PAA-ACC hydrogel to different strains was estimated from gauge factor (GF = (ΔR/R0)/ε) relation which was found to be 1.51 in the strain range of 0.3–30% and 10.79 in the strain range of 30–450%. Upon various stretching conditions, the electron path could be enlarged, leading to increased resistance. The sensor was used to monitor large-scale human motions as the sensor was attached to different joints such as an elbow, finger and even throat. It also could distinguish the radial artery pressure characteristic peaks by connecting it to a human wrist. The sensor demonstrated an augmentation index of 0.53, smaller than the statistical values (0.55), which displayed the more elastic artery sensing performance of the sensor. The augmentation index is the ratio of systolic to diastolic peaks as radial artery pressure peaks. In addition, the upstroke was calculated to be 150 ms compared to defined statistical values of 180 ms for a healthy person (Figure 9h–k). The biocompatibility and noncytotoxicity of the MXene-PAA-ACC cytocompatibility was tested by using L929 cells cultured for up to 3 days, and the cell viability was proved by high density and well distributed L929 cells, which illustrates its potential applicability as wearable on-skin epidermal sensors. Moreover, its degradation in PBS solution makes no electronic waste as a nonpolluting option [86].

In a study of Ti_3_C_2_Tx-MXene application in a wearable, breathable bioelectronic interface, Sharifuzzaman and co-workers utilized a difluoroethylene (PDFE)-based nanofiber, reinforced by Ti3C2Tx-MXene and a dehydrofluorination process to make laser-induced hierarchical CNFs (LIHCNFs). This cost-effective interface was used as an electronic tattoo to measure electrocardiography (ECG) and electroencephalogram (EEG) signals. ECG signals were recorded with sensing body movement using an electromechanical vibrator fixed on the skin of a volunteer, and EEG signals were monitored from the frontal lobe with two LIHCNFs tattoos attached to the forehead. The LIHCNFs tattoos revealed low resistance of 4 Ω sq^−1^ with impedance as low as 23.59 kΩ cm^2^ at 10 Hz compared to dry electrodes such as graphitized electrospun fiber [87], Au thin film/polyimide [88] or laser-induced porous graphene [89]. The LIHCNFs tattoos also have high signal to noise ratio of 41 dB and are suitable for long-term monitoring, revealing high durability even after 24h use [90]. The proposed bioelectronic tattoo is a potential candidate for the development of wearable human-machine biointerfaces.

Solution processed methods have attracted much more attention due to their scalability which enables development of wearable technologies. Driscoll and co-workers fabricated highly conductive Ti_3_C_2_ by facile the solution-processing method to construct an implantable multichannel neuroelectronic device with four times less impedance and in vivo neural signal sensing compared to gold microelectrodes due to the high conductivity and high surface area of MXene [91]. These neural electrode arrays enable neural recording from the cerebral cortex as well as deeper brain parts of anesthetized rats, with much higher sensitivity and lower noise in recording larger amounts of neural spikes. In terms of the biocompatibility, the Ti_3_C_2_ neuroelectronic device was tested by infusing it with neuron cultures, which resulted in no alteration or intervention with normal neural functional processes such as synapse formation or neurite viability [91]. The same group used prepared water-based Ti_3_C_2_ MXene ink and placed it on laser-patterned electrode arrays with different geometries of planar and 3D mini-pillar with no required conductive gels for epidermal electronic usage. The bulk conductivity of the MXene composites was measured to be 3015 ± 333 S/m (from 500 μm) to 241.4 ± 14.7 ohms (for 3 mm thickness) at 10 Hz. The human epidermal sensing was conducted over the inferior parietal cortex to record a high-resolution EEG as an alpha signature through dense arrays. Moreover, the electrodes were tested on hand motor area and recorded suppression of the 8 to 12 Hz motor mu rhythm as an EEG sign. The electrode demonstrated reduced electrode-skin interface impedance compared to industrial carbon conductive ink. These experiments show that the flexible bioelectronic interfaces, named as MXtrode arrays, have potential applicability in neuromuscular diagnostics and rehabilitation [92]. In the same content, Murphy and co-workers used MXene encapsulated in parylene-C gel-free in high-density EMG arrays. The impedance of electrodes in contact with human skin was recorded 100–1000 times lower than commercially available electrodes [93]. Therefore, compared with conventional metal nanostructures as metallic contact electrodes that need sintering and optimization of a thermal treatment, 2D MXene ink displays more flexibility, electrical conductivity with lower skin–electrode interface impedance and gel-free application capability which makes it a key material for future biomedical applications. 

### 5.2. Imaging

One fascinating and broad use of 2D nanomaterials is their implementation in the imaging technologies such as magnetic resonance imaging (MRI), X-Ray computed tomography imaging (CT imaging), optical imaging (OI) and PA imaging for diagnosing various diseases. It is reported that among various available 2DNMS, MOFs are promising materials for imaging applications due to their unique characteristics such as diverse compositions, high porosity, simplicity of their multifunctionalization and stability in physiological environments which enables their use as imaging contrast agents or imaging contrast carriers [94,95]. Recent research has revealed that through incorporation of Fe, Mn, Gd, iron oxide and derivatives into MOFs, specific types of nanocomposites can be generated that serve as contrast agents in MRIs and enhanced high-resolution MRIs. In addition, encapsulating superparamagnetic NPs into the MOFs makes them an appropriate nanocomposite as contrast agents [96]. Lin and co-workers decorated MOFs with Gd^3+^ for image contrast enhancement. In this study, Gd(BDC)_1.5_(H_2_O)_2_, nanorods with 40 nm in diameter and 100 nm in length demonstrated the improvement of image contrast by increasing water proton relaxation rates for MRI imaging [97]. For CT imaging purposes, elements with high Z numbers such as barium, bismuth and iodine are used as contrast agents. However, many disadvantages such as large doses requirements in order to have satisfactory contrast and inadequate distribution have limited their practical applications [98]. In this regard, 2DNMs (e.g., MOF-based platforms) as next generation contrast agents can be introduced as an appropriate alternative to overcome these restrictions and contrast enhancement. For example, Shang and co-workers synthesized Au@MIL-88(A), the core-shell gold nanorod@MOF nanoprobes via tunable growth of a MOF shell layer on the surface of gold nanorod for multimodality diagnosis of glioma. This star-like nanocomposite with an average diameter of 89 ± 3 nm exhibited high contrast efficiency in CT imaging as well as in MRI and PAI imaging [99]. In addition to the abovementioned bio-imaging technologies, MOFs have shown high potential in the optical imaging field and there has been extensive research to fabricate MOFs with sufficient luminescence as bio-imaging agents. Lin and co-workers designed the phosphorescent MOF by [Ru{5,5′-(CO_2_)_2_-bpy}(bpy)_2_] as a bridging ligand (bpy is 2,2′-bipyridine) and Zn^2+^ or Zr^4+^ as connecting points in which the zirconium MOF coated by silica and functionalized with PEG, targeted the cancer cells for in vitro optical imaging. Their findings show that the prepared nanocomposite is an efficient contrast optical imaging agent with extremely high dye loadings suitable for optical imaging [100].

Another fascinating 2D nanoplatform which has drawn considerable attention in biomedical applications is TMDs. Considering intriguing attributes such as relatively large surface-to-volume ratio allowing maximal interaction with target biomaterial and consequently enhanced efficiency and sensitivity, high stability in different environments, low toxicity, nonhazardous nature and desirable optical properties, TMDs are reported as a novel material for biomedical technologies [101]. Strong NIR absorbance, noticeable rate of light-to-heat transformation and the next generation of ultrasound signal meet requirements for application in PA imaging [102]. Chen and co-workers synthesized TMD-based nanosheets with different layers (single-layer (S-MoS_2_), few-layer (F-MoS_2_) and multi-layer (M-MoS_2_)) using albumin-assisted exfoliation without further surface modifications with potential application in PA imaging [103]. It is reported that the number of layers in these MoS_2_ nanosheets can significantly influence their PA effect. They reveal that reducing the number of layers in the nanosheets from M-MoS_2_ to S-MoS_2_ can result in outstanding enhancement in NIR absorbance, improvement in the elastic properties and excellent biocompatibility and production of a reinforced PA signal. These favorable features offer benefits of exploitation of single-layered MoS_2_ in PA imaging probes. In vitro experiments of the prepared MoS_2_ nanosheets indicate that intravenous injection of S-MoS_2_ to U87 glioma cells of mice results in significantly efficient brain tumor cell detection. Moreover, the high atomic number and the excellent X-ray attenuation capability of transition metal in TMD nanostructures make them a desirable substitute in CT imaging techniques as contrast agents [104]. Yin and co-workers designed the chitosan functionalized MoS_2_ (MoS_2_-CS) nanosheets as a contrast agent of CT imaging. These chitosan modified MoS_2_ nanosheets demonstrated prominent signal enhancement with an increase in the concentration of the agents in CT images of mice [105].

**Figure 10:**
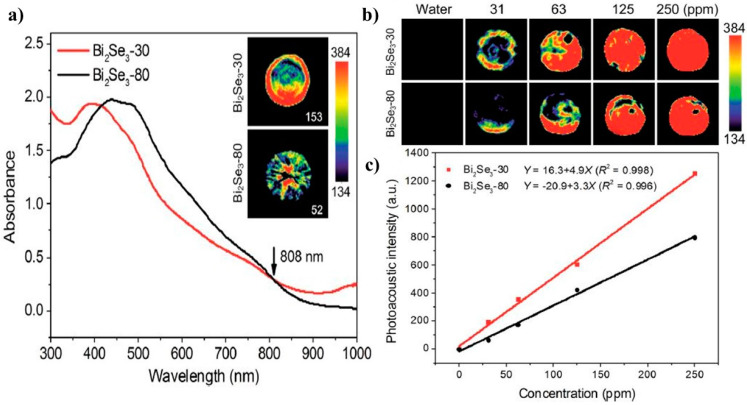
Imaging applications of 2DNMS: (**a**) photoacoustic images of the two Bi_2_Se_3_ nanosheets (30 and 80 nm) with same absorbance intensities of 0.3 at 808 nm; (**b**) photoacoustic images of the two Bi_2_Se_3_ nanosheets in aqueous solution with different concentrations from 0 to 250 ppm; (**c**) evaluation of NIR photoacoustic performance of the two Bi_2_Se_3_ nanosheets in form of studies of photoacoustic signal intensities in different concentrations (Reproduced with permission from Ref. [106]).

Another class of highly potential 2D nanomaterials in imaging technologies is topological insulators (TIs). Among different type of 2D TIs, Bi_2_Se_3_ nanosheets due to their high NIR absorption, bioactivity and biocompatibility have become more prominent in biomedical applications such as bioimaging. Xie and co-workers have proposed administration of two Bi_2_Se_3_ nanosheets with different sizes (30 and 80 nm) for optical diagnostic and photothermal therapy [106]. Their findings suggest that both Bi_2_Se_3_ nanosheets have favorable performance in all investigated features such as photoacoustic effect, optical absorption and NIR photothermal. Nevertheless, the smaller one (Bi_2_Se_3_ with 30 nm size) shows better performance which makes it a more suitable candidate for bioimaging techniques (Figure 10).

### 5.3. Drug Delivery

2DNMs have shown a great potential for drug delivery applications. Their layered structure, which enables high surface-area-to-mass ratio, adequate cell intake and routes of chemical functionalization, allows them serve as platforms of drug delivery in various therapies such as chemotherapy [69,107]. The planar topology of 2DNMs, along with their ultrathin structure, offer benefits of enhancing drug delivery performance due to their large surface area. In fact, by creating anchoring sites for antitumor and therapeutic drug molecules, this property can lead to increasing loading efficiencies of drug carriers. These desirable features attributed to 2D nanostructures employed as nanoplatforms in drug delivery are not limited to possessing high capacity of drug release; low toxicity and facile surface modification are other fascinating characteristics of these materials resulting in ever-increasing interest and efforts to develop them for drug delivery applications [2,108,109,110].

**Figure 11:**
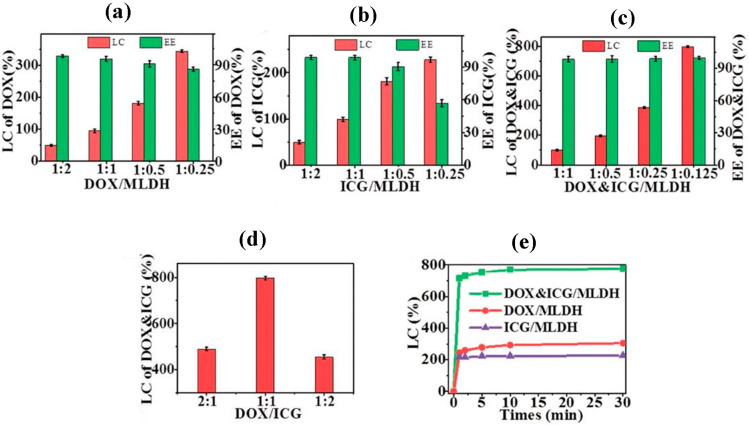
2DNMs as a unique platform for drug loading and delivery. LC and EE of: (**a**) DOX; (**b**) ICG; (**c**) DOX&ICG onto MLDH nanosheets with various mass ratios; (**d**) LC of DOX&ICG onto MLDH nanosheets with various mass ratios of DOX:IC; (**e**) adsorption curves of DOX, ICG, and DOX&ICG onto MLDH nanosheets (Reprinted with permission from ref. [112]. 2018, Wiley VCH).

One of the 2D nanostructures being evaluated in drug delivery nanosystems is layered double hydroxides (LDHs) nanosheets. Indeed, considering the results of several recent studies, these nanosheets exhibit superb performance in cancer therapies as nanocarriers. Possessing some exceptional characteristics such as the capability to be absorbed by some certain cancer cells resulting in then reduction of the possibility of endosmal effects, desirable performance in pH-responsive drug release because of high interlayer anion exchange and charge density, facile adsorption for drug molecules with negative charges, anionic antibodies and biological molecules due to the positive charge on the surface of these nanosheets and statistically significant low toxicity make them a rising star in drug delivery applications [2,69]. For instance, recently Peng and his co-workers reported the fabrication of Gd-doped LDH nanosheets through a bottom-up process that exhibit a promising advance in drug delivery and cancer therapies by nanocarriers [111]. These LDH nanosheets as nanoplatforms demonstrated extraordinary efficiency in drug loading of DOX and ICG. Compared to other reported 2D drug nanocarriers, these MLDHs hold first place in their capacity to drug load content (LC) with the highest LC level (797.36%) at almost 100% of encapsulation efficiency (EE = 99.67%) (Figure 11). Results of this study also revealed favorable behavior of DOX&ICG/MLDH composites in pH trigged and NIR responsive drug release as well as their excellent biocompatibility, which allows them to be considered as a strong candidate to improve the efficiency of drug delivery processes and then anticancer activity. 

**Figure 12:**
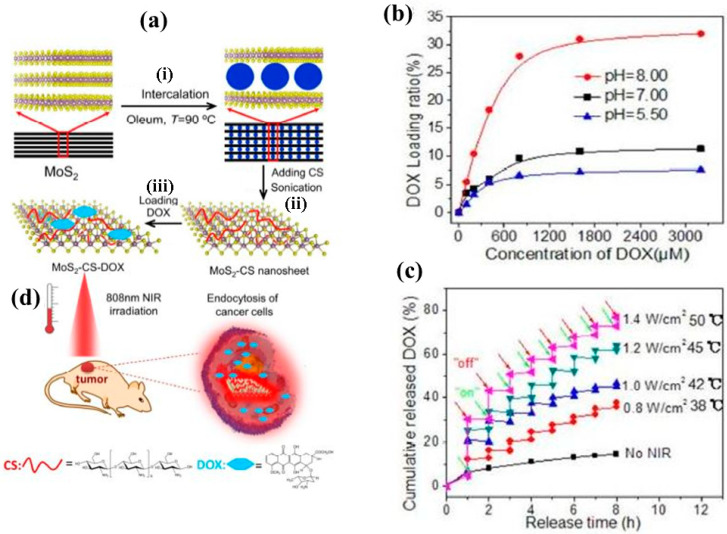
2DNMs in photothermal cancer therapy: (**a**) schematic illustration of synthesis of chitosan-modified MoS_2_ nanosheets as a NIR photothermal-triggered drug delivery nanoplatform for desirable cancer therapy: (**a**,**b**) production procedure of MoS_2_-CS via intercalation-exfoliation process of single-layer MoS_2_ nanosheets and then modification by CS; (**b**) study of loading ratio of DOX onto MoS_2_-CS nanosheets as the function of the concentration of DOX at different pH values; (**c**) assessment of the release profile of DOX in PBS buffer (pH 5.00) by considering the absence and presence of 808 nm NIR laser; (**c**) loading process of DOX as drug; (**d**) NIR photothermal-triggered drug delivery of the MoS_2_ nanosheets to tumor tissue; (Reprinted with permission from ref. [105]. 2014, American Chemical Society).

As another example of 2DNMs, TMDs have shown to be a promising 2D drug delivery platform for efficient cancer treatment. In this regard, MoS_2_ nanosheets as the most typical form of TMDs in nano-based drug delivery systems have attracted much attention. Yin and his co-workers demonstrated the possibility of efficient chemotherapy via application of functionalized MoS_2_ nanosheets with chitosan (CS) as drug nanocarriers [105]. CS-decorated MoS_2_ nanosheets were prepared by a modified intercalation-exfoliation procedure, followed by chitosan decoration to enhance the biocompatibility and physiological stability of these nanosheets. In this research, these MoS_2_-CS nanosheets met the requirements of exploitation for a NIR light-triggered drug delivery system because of strong NIR photothermal absorbance. Furthermore, the 2D planar topology of these nanostructures empowers them to access high antitumor drug (i.e., DOX) loading capacity. Results of this study also indicate favorable efficiency of DOX release of MoS_2_-CS-DOX undergone 808 nm NIR laser irradiation on account of the photothermal effect (Figure 12). 

To address health concerns related to the exploration of MoS_2_-CS nanosheets for therapeutic process, toxicity of these proposed nanocarriers for cancer therapy has been investigated. Low cytotoxicity and hemolytic activity of red blood cells in the presence of these nanosheets proved promising biocompatibility of MoS_2_-CS. Taken together, easy drug loading, controllable release after exposure to a NIR laser, low toxicity and desirable biocompatibility make these modified TMD nanosheets an ideal alternative in NIR photothermal-triggered drug delivery systems for killing tumors.

### 5.4. Tissue Engineering

Tissue engineering is an intellectual alternative to restore deprived functions of various body tissues. 2D-based materials have outperformed other materials due to their unique properties such as superior electrical and mechanical properties, biocompatibility and the capability for functionalization. To assign proper biocompatible material, it is vital to pinpoint desired features related to the specific tissue and find the best material that fulfills those expectations. The selected material that easily adopts and mimic the biological medium while maintaining its original function is then considered for that specific tissue construction [113].

**Figure 13:**
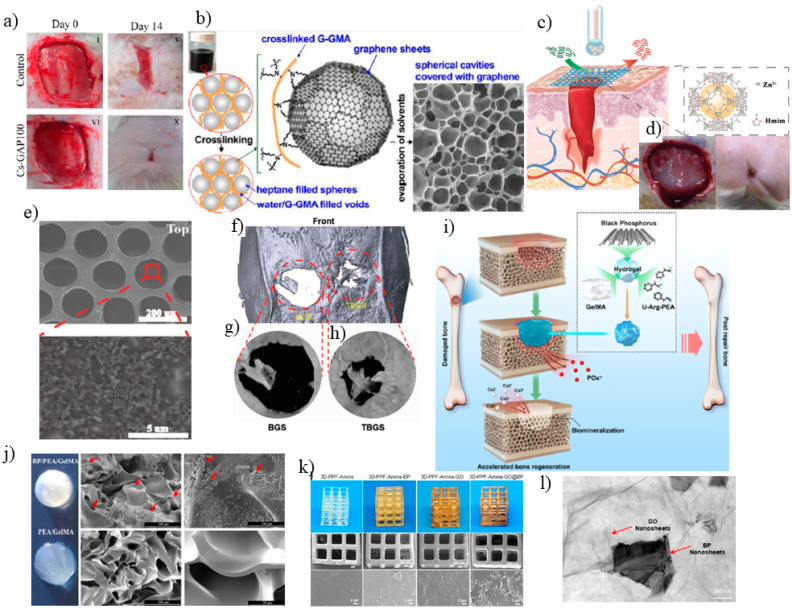
2DNMs for wound healing applications. (**a**) In vivo wound-healing images of cotton gauge (i,v), and Cs-GAP100 (vi,x) nanobiocomposite film (Reprinted with permission from ref. [123]. 2020, American Chemical Society), (**b**) Exfoliation of graphite to graphene sheets in the heptane/G-GMA containing water emulsion and Illustrations of core−skirt architecture of a Boston keratoprosthesis (B-KPro) with a typical SEM image of saturable, porous hybrid hydrogel with graphene sheets covering the lining of the spherical cavities (Reprinted with permission from ref. [125]. 2021, American Chemical Society), (**c**) Schematic showing the fabrication of the ZIF-8-laden omniphobic hydrogel membranes by a microfluidic approach and the application of resultant membranes for wound healing, (**d**) photos of the wounds following surgery (0 days), and after11 days of treatment by 500, and 5000 µg mL^−1^ ZIF-8@PVA omniphobic hydrogel membranes, (**e**) SEM images showing the porous structure from a top view, and ZIF-8 inside the membranes (Reprinted with permission from ref. [129]. 2020, Wiley-VCH), (**f**) 3D reconstruction of circular defects and (**g**,**h**) Micro-CT images of cranial defect areas (5 mm diameter) at 24 weeks after BGS and TBGS implantation showing the better in vivo osteogenesis performance of TBGS (Reproduced with permission from Ref. [130]. 2019, Wiley-VCH), (**i**) 3-D schematic of BP/PEA/GelMA Hydrogel Platform with encapsulated BPNs to enhance Bone Regeneration through Capturing Calcium Ions, (**j**) SEM images of PEA/GelMA and BP/PEA/GelMA hydrogels after 15 days of mineralization with red indicator mineral formation (Reprinted with permission from ref. [131]. 2019, American Chemical Society), (**k**) TEM image of BP nanosheets encapsulated GO nanosheets, and (**l**) SEM images of varied morphologies of BP and GO on the surfaces of the 3D-PPFAmine-GO@BP scaffold (Reprinted with permission from ref. [132]. 2019, American Chemical Society).

Graphene, due to its electrical conductivity, hardness, biodegradability and flexibility is one of the most well-known 2D materials in tissue engineering and implants [114]. Biocompatibility, flexibility and antibacterial properties of graphene-based 2D materials can provide a scaffold for artificial neural tissue engineering for nerve regeneration [115], articular cartilage tissue engineering [116] and prevention of bacterial growth in dental tissues [117]. Moreover, based on its mechanical stability, it can be utilized in tissues that require stiffness such as bone-tissue engineering. It can be combined with hydrogels [118], fibers [119], polymers [120] and other scaffolds to reinforce their mechanical properties. Kolanthai and co-workers prepared alginate−chitosan−collagen-graphene oxide (SA–CS–Col–GO) composite scaffold by freeze-drying and ionically crosslinking with calcium ions. Compared to scaffolds in which the microporous structure is not refined with GO nanosheets, SA–CS–Col–GO demonstrated better mechanical property with increased modulus to 0.87 ± 0.05 MPa at 40% strain, due to the hydrogen bond of GO (–OH and –COOH functional groups) with the hydroxyl group of SA. The SA–CS–Col–GO showed improved mouse osteoblast cell growth with a higher number of live cells in comparison to other scaffolds, suggesting that the GO incorporated scaffold can boost the osteoblast cell proliferation and reveal better biocompatibility. Moreover, the SA–CS–Col–GO scaffold gained more stability in water compared to non-GO filled samples and the swelling ratio was significantly decreased in water (pH 7) and PBS (pH 7.4) due to increased crosslinking in the presence of GO [121]. Another study by Li and co-workers was conducted by preparing a three-dimensional GO foam/polydimethylsiloxane/zinc silicate (GF/PDMS/ZS) composite scaffold through dip coating and hydrothermal synthesis method, to make a macroporous platform for bone-tissue engineering [122]. The mechanical strength of the composite was enforced by incorporating PDMS to GF because the compressive modulus increased. Furthermore, the porosity of GF/PDMS/ZS composite measured 87.35% compared to GF/PDMS scaffold with 70.16%, an important factor in bone tissue engineering. In vitro studies using laser confocal images of cells cocultured with GF/PDMS/ZS scaffold after 7 days revealed more mouse bone marrow mesenchymal stem cells (mBMSCs) grew than on GF, GF/PDMS scaffolds and expression of alkaline phosphatase (ALP) and runt-related transcription factor 2 (RUNX-2) gens as markers of osteogenic differentiation were enhanced. For in vivo analysis, rabbits’ bone defects were treated with the GF/PDMS/ZS and revealed comparable bone formation after 12 weeks of implantation with no inflammatory reaction [122]. Incorporation of a graphene-silver-polycationic peptide (GAP) nanocomposite into chitosan (Cs) in the form of sponge can also provide a scaffold for wound healing [123]. Graphene with high surface area strengthens the scaffold with maximum tensile strength of 58.33 ± 1.99 MPa with increasing nanocomposite concentration due to the π−π interaction of graphene with the chitosan structure and enhances the stability. Hemocompatibility of the scaffold was not more than 2.9% hemolysis. Silver NPs and polycationic peptide induce an antimicrobial scaffold against *Escherichia coli* and *Staphylococcus aureus* [124]. The hydrophilic structure of a sponge scaffold due to its graphene-polycationic peptide nanocomposite decreases the blood clotting time to 60 s. In vivo wound-healing efficacy of a nanobiocomposite scaffold was studied using a Wistar rat model. After 14 days, the Cs-GAP100 nanobiocomposite film-treated rat group showed faster and complete healing, with the wound closure of 97.12 ± 2.65% compared to the control group (Figure 13a) [123]. In another study by Sharifi and co-workers, to enhance gelatin glycidyl methacrylate hydrogel, graphene-coated microspherical cavities were introduced in the structure by reverse solvent interface trapping method to make microspherical cavities covered by exfoliated graphene (Figure 13). The biocompatibility of the hybrid was tested by live-dead assays that demonstrated viability of more than 90% after maximum 7 days of cell culture compared to cells grown on a tissue culture well plate (TCP) positive control (Figure 13b) [125].

MOFs containing transition metal ions interconnected with organic ligands through hydrogen bonding or other electrostatic interactions can be applied to implants to help wound healing. Recently, 3D printing combined with MOFs [126], polymeric nanocomposites of MOFs [127] and natural mineral-based MOFs [128] were utilized for tissue engineering. Yao and co-workers prepared an omniphobic porous hydrogel wound dressing loaded with a zinc imidazolate framework 8 (ZIF-8@PVA) via a microfluidic emulsion templating technique to reduce bacterial contamination, while providing the wound with optimized zinc ion release to target bacteria. It was found optimized 500 μg mL^−1^ ZIF-8@PVA improves wound closure after 11 days due to its cytocompatibility (Figure 13c–e) [129]. MXenes can also be applied as biomaterials for tissue engineering, cellular growth and osteogenesis differentiation of bone marrow derived mesenchymal stem cells (BMSCs). In a study doped Ti_3_C_2_ MXene composite nanofibers were obtained via electrospinning showing hydrophilicity due to the functional groups on the surface [17]. Cellular fluorescent images of MXene composite nanofibers after 5 days of culture revealed uniform cellular spread compared to a control group. Reverse transcription polymerase chain reaction (RT-PCR) analysis was employed to evaluate osteogenic differentiation performance by detecting the marker genes, and the results of cells cultured on MXene composite nanofibers after 14 days of culture demonstrated much better osteogenic differentiation than that on control groups [17]. Pan and co-workers used Ti_3_C_2_ MXene to kill bone tumor as well as expedite bone tissue regeneration in the presence of composite 3D-printing bioactive glass (BG) scaffolds in vivo [130]. Observation and microcomputed tomography (micro-CT) analysis of Ti_3_C_2_-BG scaffold (TBGS) implants in Sprague–Dawley rats after 24 weeks displayed boosted bone-tissue regeneration, which confirmed better osteogenic performance of TBGS compared to the bare one (Figure 13f–h). The long-term toxicity of implanted bone defects was also tested after 24 weeks by collecting venous blood from major organs of rats and demonstrated no alteration compared to the control group [130].

Black phosphorus nanosheet (BPN)-based hydrogel was prepared by cross-linking gelatin methacrylamide, BPNs and cationic arginine-based unsaturated poly (ester amide)s (BP/PEA/GelMA hydrogel) in which BPNs can degrade into phosphorus ions and capture calcium ions to regenerate bone defect in vivo [131]. In vitro investigation of mineralization capacity was conducted by immersing BP-modified hydrogel in body fluid under natural light for 15 days, which showed white mineralization in SEM images (Figure 13i). It was found that BPN-containing hydrogels improved osteogenic differentiation of human dental pulp stem cells, where the protein levels of Col-1, BMP4, and RUNX2 were significantly greater for BPN-containing hydrogels compared to other hydrogels after 14 days of incubation. Moreover, phosphorus-rich hydrogels also enhanced bone regeneration in vivo [131]. 

The enhancement of cell proliferation and osteogenesis was also reported by Liu and co-workers through synergic hybrid of 2D black phosphorus and graphene oxide in a 3D printed poly (propylene fumarate) scaffold (3D-PPF-Amine-GO@BP scaffold) [132]. Immunofluorescence observation by confocal imaging demonstrated increased cell proliferation on 3D-PPF-Amine-GO@BP hybrid scaffold with higher cell density after 6 days of culture. In fact, the wrapped BP in GO nanosheets allow slow oxidation of BP which continuously releases phosphate ions as an osteoblast differentiation facilitator. The gradual release of phosphate ions demonstrated an increase in mineralization with calcium phosphate NPs compared to bare 3D scaffold (Figure 13k,l). Therefore, in 3D-PPF-Amine-GO@BP scaffold each part plays its role to improve mineralization, phosphate ion generation, biocompatibility, and osteogenic capacity of the whole scaffold. GO nanosheets with large surface area helped improve protein and cell adhesion; BP nanosheets allowed for continuous phosphate release from scaffolds; BP and GO nanosheets synergetic correlation also led to highest cell proliferation and osteogenesis properties [132]. All in all, 2D materials seems to help improve loading capacity, biocompatibility and osteogenesis properties compared to conventional nanoparticles in scaffolds.

### 5.5. Photothermal Therapy

**Figure 14:**
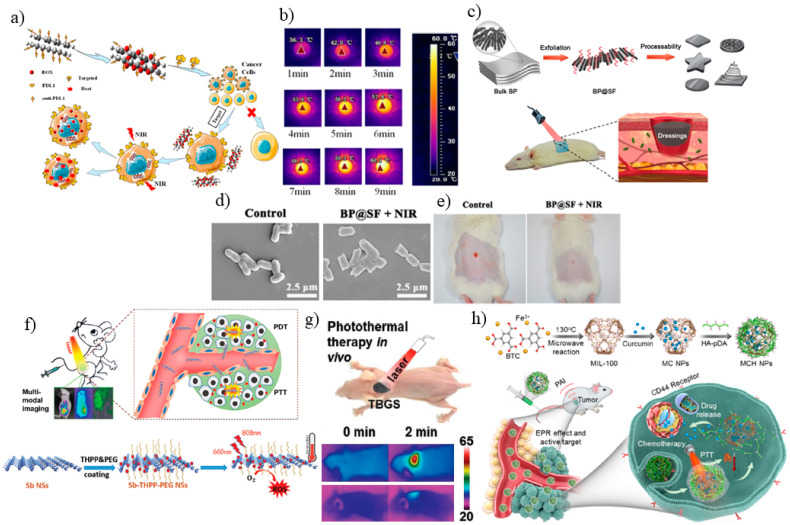
Photothermal therapeutic applications of 2DNMs: (**a**) schematic of TPD application for targeted chemophotothermal therapy; (**b**) NIR thermal images of TPG at the highest temperature at various times (Reprinted with permission from ref. [137]. 2020, American Chemical Society); (**c**) schematic of the fabrication process of the BP@SF and its application for wound healing of mouse skin; (**d**) morphology of the bacteria after BP@SF treatment with NIR irradiation; (**e**) in vivo PTT photographs of *E. coli* infected skin wounds after BP@SF treatment with NIR irradiation (Reprinted with permission from ref. [138]. 2018, American Chemical Society); (**f**) schematic diagram of modification method of Sb NSs and light therapy of mice under multiple imaging guidelines (Reprinted with permission from ref. [139]. 2021, Wiley VCH); (**g**) schematic illustration of in vivo photothermal cancer ablation with TBGSs implantation and IR thermal images at BGS-implanted tumor (bottom) and TBGS-implanted tumor (top) sites of Saos-2 tumor-bearing mice under laser irradiation (Reprinted with permission from ref. [130]. 2020, Wiley VCH); (**h**) preparation process of MCH NPs and using MCH NPs for PAI-guided chemo/photothermal combinational tumor therapy (Reprinted with permission from ref. [142]. 2018, American Chemical Society).

Photothermal therapy (PTT) is a non-invasive therapeutic technique with the least side effects compared to conventional chemotherapy and radiotherapy methods. In PTT, NIR laser deeply enter tissue and will be absorbed in photothermal agents to locally heat up cells. The electrons in PTT agents are excited to higher states and after relaxation make high kinetic energies to heat and destroy cancer cells. To obtain more light-to-heat efficiency and less destructive performance, the PTT agents should demonstrate high absorption cross section. This milestone has been mostly achieved by noble metal NPs compared to dye photo-absorbers, due to strong surface plasmon resonance (SPR) and photostability. Gold and silver nanostructures [133] and carbon nanomaterials [134] are well- studied as contrast agents for thermal ablation of cancer cells [135]. However, 2DNMs are more versatile due to changeable layered structure which allows surface modification with NPs and molecules [136]. In a recent study, reduced graphene oxide (rGO) with tea polyphenol (TPG) as one of the well-known 2D nanoplatforms used for targeted photochemotherapy and it was found that the therapeutic efficacy increases with programed cell death-ligand 1 (PDL1) modification [137]. The results revealed that pure doxorubicin hydrochloride (DOX) cancer drug shows a strong cancer and normal cells ablation, but when loaded on anti-PDL1-conjugated TPG (TPDL1) with 1:100 PDL1/TPG ratio, as chemotherapy agent, and TPDL1 as a targeted photo-chemo-thermal agent with near-infrared (NIR) irradiation increases anticancer drug release efficiency while doing no harm to normal cells (Figure 14a). In fact, DOX releasing activity suppressed from 22.60% to 5.31% when pH changes from 5.0 to biological pH = 7.4 which allows TPD to circulate with blood easily and releasing in the acidic environment of cancer cells. TPG at different concentrations revealed no obvious cytotoxicity in 72 h to normal cells (L929 and PDLCs) through CCK- while allowing targeted killing of CAL-27 and ACC2 cancer cells when its concentration reaches 250 μg·mL^−1^ due to generation of reactive oxygen species (ROS) inside CAL-27 when TPG is added. TPG also revealed reversible photothermal conversion when it was irradiated with the NIR laser for time duration of 3 min (on/off cycle) as a proof of its stability. However, functionalization of TPG with the chemotherapy agents allowed using lower concentrations of TPG which makes it less aggregated in cells. In PTT test, addition of TPD (1 μM DOX) enhances the temperature to about 55 °C in 5 min (Figure 14b), with enhanced anticancer effect of 71.57%, higher than that of TPG (57.51%), and TPDL1 (55.99%) due to NIR treatment enhance ROS generation [137]. As a result, in comparison to pure cancer drugs, rGO along with drug can help to suppress side effects, while enhancing cancer cells cytotoxicity.

2DNMs can also be utilized for wound healing with antibacterial properties. In a study by Huang and co-workers, an ultrasonication-assisted liquid exfoliation technique was adopted to generate highly stable black phosphorous (BP) nanosheets using silk fibroin (SF) as an exfoliating agent for wound dressing (Figure 14c) [138]. SF modified-BP (BP@SF) antibacterial activity was tested with E. coli (Gram-negative) and B. subtilis (Gram-positive) bacteria as representative models. The bacteria cocultured plate with BP@SF was irradiated with NIR light followed by plate counting. Both bacteria’s viability reduced compared to applying either BP@SF dressing or NIR laser irradiation. The viability assay with confocal fluorescence images of E. coli or B. subtilis cells was also performed, where most E. coli and B. subtilis cells were found dead after NIR irradiation. SEM images of bacteria revealed a distorted appearance after irradiation, confirming the PTT performance of our BP@SF dressing in killing bacteria (Figure 14d). The in vivo PTT results demonstrate wound repair of mice tissue after 5 days (Figure 14e) and the residual bacteria of skin treated with the PTT agent was tested through measuring their optical density (OD_600_), and it was found that the OD value of BP@SF alone was higher than that of control sample since BP@SF dressing on the wound might partially produce phosphate or phosphonate after degradation. Moreover, no abnormality was detected in mice organs when some parts were sliced for hematoxylin and eosin (H&E) histological analysis. Therefore, the BP@SF dressing using silk fibroin as an exfoliating and stabilizer agent was able to effectively prevent bacterial infection and improve wound repair [138]. 

To increase the NIR-light-to-heat conversion efficiency of 2D nanomaterials, Kang and co-workers loaded photosensitizer 5,10,15,20-Tetrakis(4-hydroxy-phenyl)-21H,12H-porphine (THPP) at the surface of antimonene nanosheets (Sb NSs) followed by poly(ethylene glycol) (PEG) modification [139]. This modification allowed photothermal conversion efficiency of 44.6% of Sb–THPP–PEG NSs higher than most of the photothermal agents reported by others [140,141]. The Sb–THPP–PEG NSs display a Z-scheme heterojunction between Sb and THPP light absorbers due to their relevant band levels and improved charge carrier separation with generation of oxygen species ^1^O_2_ by THPP and ^●^O_2_ by Sb NSs (Figure 14f). The composite displayed high photostability when tested for a 5 min cycle (on/off) with laser (808 nm) irradiation with the highest achievable temperature of 52 °C with composite concentration of 200 μg mL^−1^. The composite material was then applied to in vitro and in vivo antitumor experiments. The biocompatibility of the composite in vitro was tested by evaluating the cytotoxicity of the composite with a cell viability examination after treatments with the composite through MTT assay standard cancer cell model of MCF-7, HeLa, 4T1, and NHDF cells. When laser triggered, more than 90% cells were killed by Sb–THPP–PEG NSs with 808 and 660 nm laser irradiations, while without applying laser, above 90% of cells remained untreated. To perform in vivo tumor treatment experiments, nanocomposites were injected in the tail vein of mice. They were irradiated under the 660 nm or/and 808 nm laser after 24 h, which resulted in raising the temperature detected by infrared imager, while no temperature rise was detected for THPP under irradiation, confirming more efficient light-to-heat conversion efficiency of the composite. The tumor growth in mice was suppressed and continued the downward trend after 10 days when the composite was applied and 660 nm plus 808 nm laser illuminated at mice with the minimal tumor size compared to either using 660 nm or 808 nm laser. For in vivo toxicity examination of Sb–THPP–PEG NSs, the mice’s serum was collected after 1, 7, and 14 days of injection, and the standard biochemistry assay, blood hematology data and H&E staining revealed no obvious inflammation and tissue ablation in the prime organs of the mice [139]. As such, antimonene nanosheets can improve light to heat conversion efficiency when applied with other photosensitive materials, while maintaining the biocompatibility and tumor degradation strength.

In the mentioned study by Pan and co-workers, Ti_3_C_2_ MXene was applied to kill bone tumor via photothermal therapy before bone-tissue engineering process [130]. Composite 3D-printing bioactive glass (BG) scaffolds were integrated with Ti3C2 NSs called TBGS to achieve higher photothermal conversion efficiency in vivo. When TBGSs were exposed to an 808 nm laser irradiation for 10 min at a power density of 1.0 W cm^−2^, the equilibrium temperature increased from 55 to 65 °C within 10 min, while the temperature of BGS in the same condition did not remarkably increase. The high photothermal stability of the MXene-integrated composite was proven by five 3 min (on/off cycles) of laser irradiation with no obvious alteration of heating curves. Furthermore, after the Saos-2 cells (osteosarcoma cells) were incubated with TBGSs and irradiated by 808 nm laser, less than 40% of Saos-2 cells survived in the TBGS + laser group, revealing the ability of TBGS for efficiently killing cancer cells by photothermal ablation. In vivo photothermal tumor ablation study of TBGS was investigated using female BALB/c nude mice bearing Saos-2 bone tumor. Under NIR laser irradiation, the surface temperature of TBGSs-implanted tumors escalated to a temperature of 63 °C within 2 min, while BGSs-implanted tumors experienced a small increase to about 37 °C (Figure 14g). Moreover, the treated TBGSs-implanted tumors permanently, while in other treated groups the tumor began to grow continuously again after treatment. This shows that composite scaffold use provides the advantage of efficient photothermal conversion of 2D Ti3C2 MXene along with bone regeneration of BG scaffolds [130].

MOF can also be utilized for loading chemotherapeutics in photothermal therapy due to their super encapsulating property. In a study by Zhang and co-workers, curcumin was loaded on the ferric ion sites of MIL-100, followed by preparing polydopamine-modified hyaluronic acid (HA-PDA)-coated MIL-100 to engineer stable MOF nanoparticles (MCH NPs) to promote photothermal conversional efficiency (Figure 14h) [139]. MCH NPs shows considerable absorbance at 808 nm compared to slight NIR absorbance of MIL-100. Therefore, MIL-100 revealed almost no temperature enhancement under 808 nm laser irradiation in 6 min, whereas after being loaded with curcumin, the MC NPs temperature reached as much as 38.9 °C, due to interaction among Fe^3+^ in MIL-100 structure and phenol groups in curcumin. The photothermal conversion efficiency of the MCH NPs was evaluated as 20.98% when PDA was applied. Since HA-PDA is detached at acidic pH, which increases drug release, it is expected that in the tumor environment, curcumin release is accelerated. The cytotoxicity of MCH NPs was evaluated in HeLa cells, CHO cells, A549 cells, and MRC-5 cells under 808 nm laser irradiation for 5 min. The cells exhibited much lower viability compared to nonirradiated cells, confirming the chemophotothermal combinational therapy capability of MCH NPs. For in vivo photothermal analysis, the MCH NPs were injected into xenograft HeLa tumor-bearing mice, where the MCH NPs accumulated at the tumor site and achieved photoacoustic imaging-guided chemo-photothermal combinational tumor therapy to accomplish tumor ablation compared to curcumin or MCH NPs tumor inhibition strength. Based on these findings, we can conclude that MOF hollow structure can be a host to accumulate chemotherapeutics and gradually release based on pH releasing mechanism in the tumor location [139].

## 6. Biosafety/Toxicity Evaluations of 2DNMs

**Figure 15:**
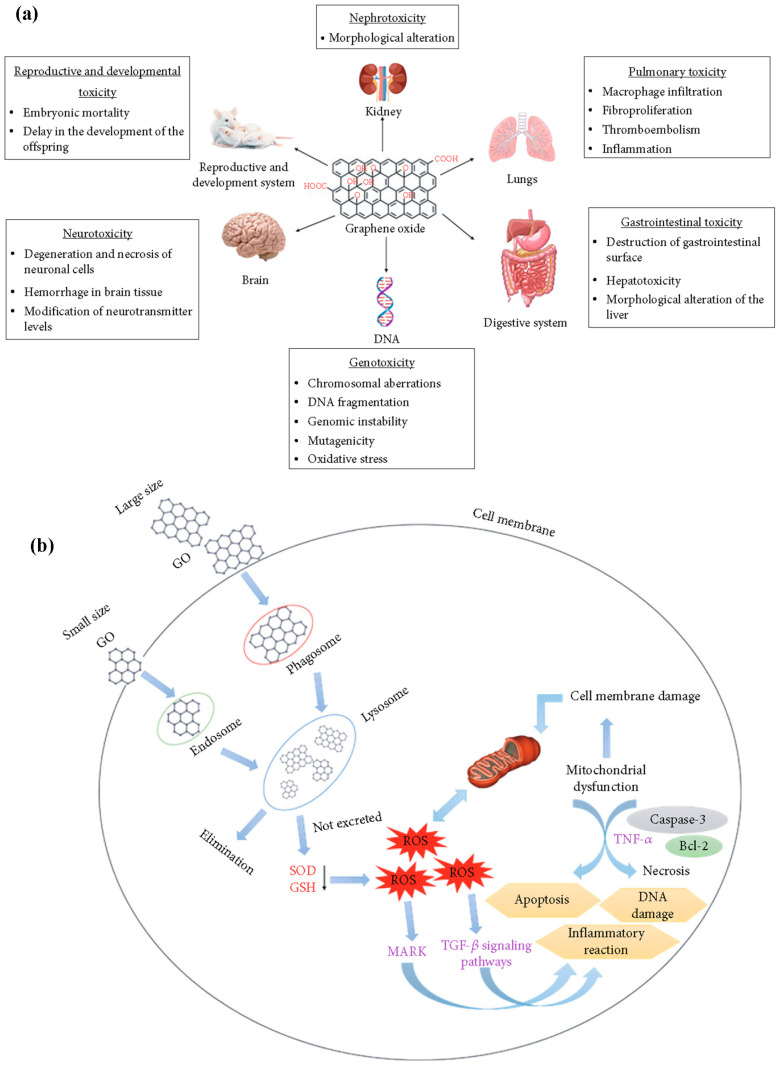
Toxicity issues of 2DNMS: (**a**) Plausible deleterious effects of GO on body organs; (**b**) schematic illustration of mechanism of GO- induced toxicity (Reprinted with permission from ref. [145]. 2021, Hindawi).

2DNMs have shown to be promising materials for future biomedical applications due to their unique physicochemical, optical, and mechanical properties. Although 2DNMs have shown a great potential in advanced biomedical technologies; (e.g., drug delivery and therapies), there is still a major failure in clinical translation of such nanomaterials. In fact, a major portion of the available studies report on superior design and higher efficacy of their nanoscale product while most of them never have a chance to pass clinical trials. Many of them fail due to numerous reasons such as toxicity and biosafety [143]. In other words, the practical biomedical applications of nanomaterials are limited by the risk of potential health issues and other risk factors. When the human body is exposed to health hazards of nanomaterials through various routes (e.g., skin contact, inhalation, ingestion, injection, etc.) they undergo some variations such as dissolution, agglomeration or formation of biomolecular corona (BC) which may contribute to different levels of toxicity [144]. For instance, some studies indicate deleterious effects of GO because of its ability to accumulate in lungs, including fibrosis, inflammatory, pulmonary edema, etc. (Figure 15) [145,146]. In fact, health concerns are one of the most critical challenges facing nanomaterials and can impede thriving advancements of these highly beneficial materials in medical applications. Therefore, there is an urgent need for design and adoption approaches to assess the potential danger of these engineered nanomaterials. 

Some features such as high surface to volume ratio (large active surface area) can result in more exposure to these manufactured nanomaterials for cells, leading to an increase in cellular interactions and consequently raising the possibility of toxicity [146]. Dutch and co-workers revealed the role of GO in observed adverse effects on lungs for more than 21 days after implementation of this material. It is reported the activation of reactive oxygen species (ROS) via GO led to the change in rate of respiration in mitochondrial, then damage of DNA and apoptosis [147].

It is also well documented that the cytotoxicity strongly depends on various physicochemical properties such as size, shape, thickness, chemical composition and surface modification of nanomaterials, and therefore, any change in these features can alter the toxicity level of nanomaterials [148]. Li and co-workers demonstrated the effect of surface oxidation density of graphene oxide on cellular damage in a murine lung [149]. Their findings indicated the amount of graphene-induced lipid peroxidation, damage to membrane, cell death and accordingly the level of cytotoxicity for GO with higher surface radical content is more than pure graphene oxide or reduced ones (rGO). Indeed, in this study the pristine GO and rGO exhibited minimal cytotoxicity. As a result, through the change in the level of surface functionality, we are able to minimize the toxicological effects and maximize biocompatibility of GO. Similar to graphene and its derivatives, TMDs may induce some cell function disturbances such as increased ROS level, cell proliferation and apoptosis, but several studies revealed that TMDs show lower cytotoxicity compared to graphene and graphene oxide [104,150]. Toxicity evaluations of different TMDs also displayed the impact of some properties such as composition and functionalization on their cytotoxicity [104]. For example, Yin and co-workers reported the role of surface modification of MoS_2_ nanosheets in the reduction of cytotoxicity [105]. Their findings showed that the modified MoS_2_ with chitosan exhibits more cell survival for two kinds of human cells in contrast to unfunctionalized MoS_2_. Therefore, functionalization with chitosan in this TMD led to reduction of TMD-induced cell death and hence more biocompatibility. Like TMDs, MXenes-based nanomaterials exhibit desirable biocompatibility [151,152]. Lin and co-workers conducted a study that revealed Ti_3_C_2_-SP nanosheets even in high doses such as 400 μg mL^−1^ demonstrate no apparent cytotoxicity [153]. Results of this research signify that regardless of MXene nanosheet concentration, viability of human cells (in this study 4T1 cell) after 48 h coincubation is still favorable and the adverse effect is insignificant.

In this regard, exploration of the toxicity of 2DNMs shows their great potential for further use in biomedical due to the low cytotoxicity. In addition, modification of some properties (such as size, surface functionality, etc.) can improve their biocompatibility by minimizing the 2DNMs-induced adverse biological effect [146]. It is noteworthy that these investigations on the emerging 2DNMs such as TMDs and MXenes are fairly new, still in progress, and need more studies to achieve comprehensive understanding of their toxicity. In particular, the focus of the recent research is mainly on short-term cytoxicity related to application of these 2DNMs, whereas other impact factors on biosafety such as dispersibility, solubility, biodegradation, immunotoxicity, genotoxicity and long-term cytotoxicity are still to be explored [151,152].

2DNMs beyond graphene have shown to have a high potential in theranostics applications due to integrated capabilities of therapeutics and diagnostics in a single nanoplatform. There is growing evidence that 2DNMs-based nanocomposites exhibit synergistic advantages in chemo, electro, and photo-therapies due to their ultrathin planar nanostructure which provides numerous anchoring sites for therapeutic drug molecules [154]. For example, it is reported that Nb_2_C MXene nanosheets show improved chemo and photo-therapy through a second near infrared (NIR) window (i.e., 1000–1350 nm) demonstrating a higher tissue penetration depth ability than the first NIR window (i.e., 750–1000 nm) [155]. Unlike conventional chemotherapies, the Nb_2_C MXene based nanoplatforms guarantee high drug-loading capacity (i.e., 32.57%) due to high active surface area. In fact, such high active surface area provides nanoengineering of 2D materials and cancer cell targeting. 

## 7. Conclusions and Future Perspectives

Following the discovery of graphene as the first 2D material, other 2DNMs beyond graphene and its derivatives such as TMDs, TIs, phosphorene, antimonene, bismuthene, MOFs and very recently MXenes attracted intensive attention due to their unique physicochemical properties compared with graphene. In this paper, we have highlighted the most recent findings and progress on biomedical applications of 2DNMs beyond graphene. We have also reviewed the most promising platforms of 2DNMS for future biomedical applications including bioelectronics, drug delivery, tissue engineering, imaging, and cancer therapy. Due to their particular optical properties, 2DNMs are promising nanoplatforms for NIR photothermal therapies. Although more systematic investigation is necessary, 2DNMs have demonstrated a high biocompatibility and suitability for tissue engineering and drug delivery applications. In addition, development of novel surface functionalization approaches facilitated production of high water-soluble 2DNMs with enhanced biocompatibility and biodistribution. The biosafety and toxicity of 2DNMs is critically important and needs to be carefully investigated before any clinical translation. More systematic studies are needed to understand the long-term toxicity of each kind of 2DNMs. Although there are many unpredicted challenges ahead in the development of 2DNMs for practical biomedical applications, the unique physicochemical properties of 2DNMs enables development of intelligent structures that are multifunctional, adaptive, programmable, and biocompatible [19].

## Data Availability

Not applicable.
